# Adult Male with Leg Swelling after a Fall Two Weeks Prior

**DOI:** 10.5811/cpcem.2017.11.36544

**Published:** 2018-01-18

**Authors:** Wirachin Hoonpongsimanont, Amal S. A Akeel, Preet K. Sahota, Shadi Lahham, Mohammad A. Helmy, Shahram Lotfipour

**Affiliations:** * University of California, Irvine, Department of Emergency Medicine, Irvine, California; † University of California, Irvine, Department of Radiological Sciences, Irvine, California

## CASE PRESENTATION

**Image 1:**
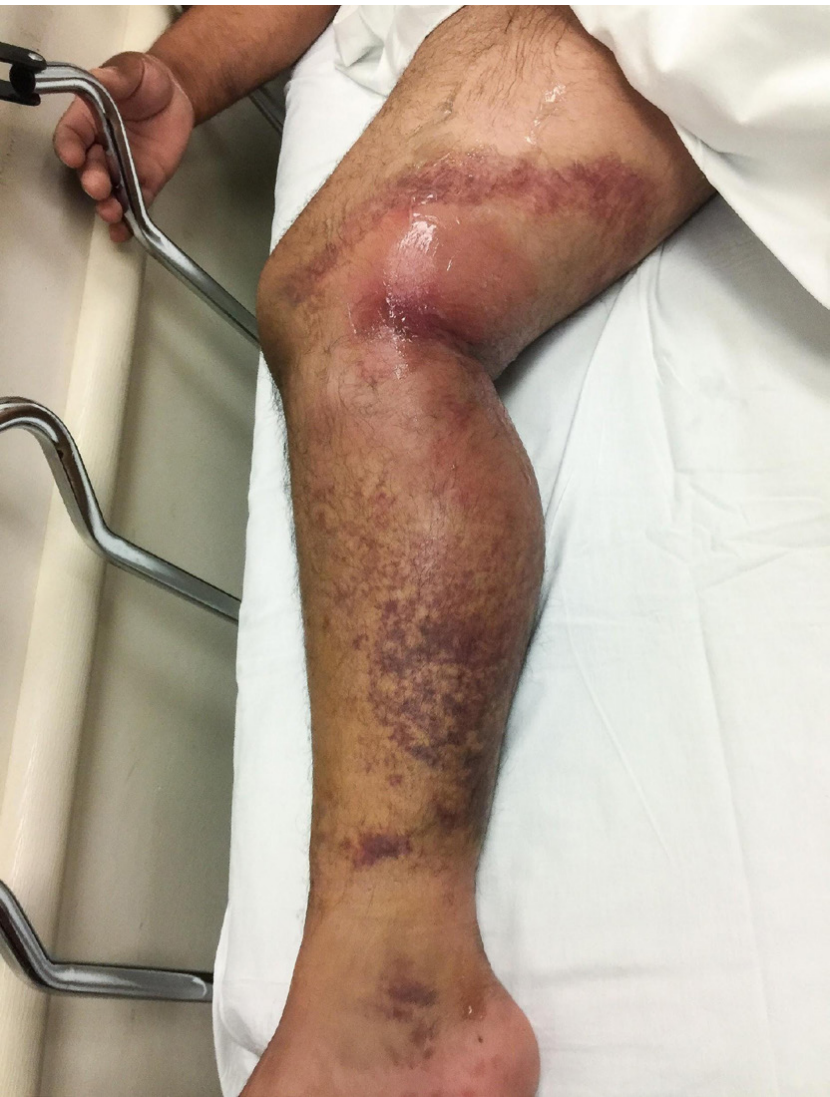
Swelling at the right knee and leg in patient thought to have early necrotizing fasciitis.

**Image 2:**
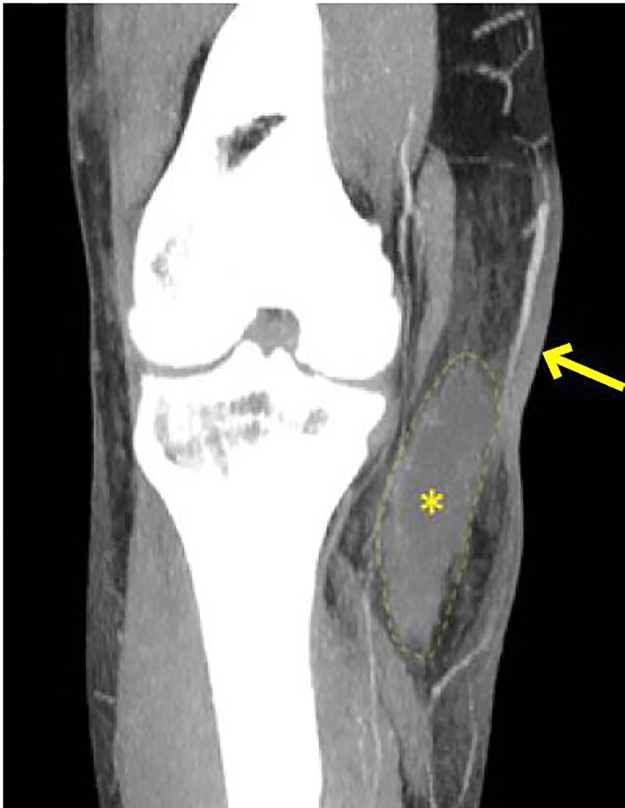
Coronal computed tomography image at level of the knee joint demonstrates an abscess (dashed outline with asterisk) in the medial subcutaneous tissues superficial to the muscle planes. The overlying skin thickening (arrow) reflects cellulitis.

A 49-year-old male presented to the emergency department (ED) with right knee pain and swelling for two days after falling from a two-story roof two weeks prior (Image 1). He visited the ED immediately after the fall; however, his radiographs showed no acute pathology. He was able to ambulate until significant swelling, bruising and pain ensued. His blood pressure was 111/71 millimeters of mercury, heart rate was 108 beats per minute, oxygen saturation was 97% at room air, and temperature was 37.4 degrees Celsius. His laboratory tests showed leukocytosis (white blood cell count: 35,500 cells per microliter), elevated c-reactive protein level (17 milligrams per deciliter (mg/dl)), lactate level (1.6 millimoles per liter (mmol/L)), sodium level (135 mmol/L), creatinine level (0.7 mg/dl), glucose level (143 mg/dl) and hemoglobin (14.9 grams per deciliter). A computed tomography (CT) of his right lower extremity was obtained (Image 2).

## DIAGNOSIS

The patient was started on intravenous clindamycin and piperacillin/tazobactam. Surgery was consulted and requested a CT. The incision and drainage was performed four hours after his presentation to the ED. The surgeon found an abscess in the medial aspect of the leg at the level of the knee above the fascial plane with healthy-appearing muscle. The patient was discharged home in good condition after four-day admission.

Given the rapid progression of the infection, an operative finding of an abscess above the fiscial plane, and a tissue culture that grew Group A *Streptococcus*, we concluded that the patient had early necrotizing fasciitis (NF). NF should be suspected even in immunocompetent hosts. Important clues are rapidly progressive violaceous lesions and a sudden onset of severe pain. A clinical staging of disease has been proposed based on cutaneous findings (Table 1).^1^,^2^

The common pretest probability tool used is the Laboratory Risk Indicator for Necrotizing Fasciitis (LRINEC) score. Our patient had a LRINEC score of six, which is associated with longer intensive care unit stay and higher septic shock and mortality rate.^3^ CT and magnetic resonance imaging are the radiological tests of choice in diagnosing NF, but obtaining these images could delay the definitive treatment.^4^,^5^ Although dissecting air along the fiscal plane is pathognomonic for NF, NF is a clinical diagnosis. Non-specific CT findings (i.e., dermal thickening, cellulitis mimics) can be found in early NF.^6^

CPC-EM Capsule

What do we already know about this clinical entity?

Necrotizing fasciitis should be suspected even in immunocompetent hosts. Important clues are rapidly progressive violaceous lesions and sudden onset of severe pain.

What is the major impact of the image(s)?

Non-specific CT findings (i.e., dermal thickening, cellulitis mimics) can be found in early necrotizing fasciitis, within the appropriate clinical presentation.

How might this improve emergency medicine practice?

Ultimately, necrotizing fasciitis is a clinical diagnosis. The provider should not solely rely on the images to establish the diagnosis and treatment.

## Figures and Tables

**Table 1:** Evolution of physical signs of necrotizing fasciitis, from early to late stages.^2^

Stage One (early)	Stage Two (intermediate)	Stage Three (late)
Warm to palpation	Blister or bullae formation (serous fluid)	Hemorrhagic bullae
Erythema	Skin fluctuance	Skin anesthesia
Tenderness to palpation (extending beyond the apparent areas of skin involvement)	Skin induration	Crepitus
Swelling		Skin necrosis with dusky discoloration progressing to frank gangrene
